# A Sensitive Response Index Selection for Rapid Assessment of Heavy Metals Toxicity to the Photosynthesis of *Chlorella pyrenoidosa* Based on Rapid Chlorophyll Fluorescence Induction Kinetics

**DOI:** 10.3390/toxics11050468

**Published:** 2023-05-19

**Authors:** Tingting Gan, Gaofang Yin, Nanjing Zhao, Xiaoxuan Tan, Ying Wang

**Affiliations:** 1 Key Laboratory of Environmental Optics and Technology, Anhui Institute of Optics and Fine Mechanics, Hefei Institutes of Physical Science, Chinese Academy of Sciences, Hefei 230031, China; ttgan@aiofm.ac.cn (T.G.); xiaox0922@163.com (X.T.); wangying@aiofm.ac.cn (Y.W.); 2 Science Island Branch of Graduate School, University of Science and Technology of China, Hefei 230026, China; 3 Key Laboratory of Optical Monitoring Technology for Environment of Anhui Province, Hefei 230031, China

**Keywords:** JIP test, heavy metal, microalgae, photosynthesis, chlorophyll fluorescence induction kinetics

## Abstract

Heavy metals as toxic pollutants have important impacts on the photosynthesis of microalgae, thus seriously threatening the normal material circulation and energy flow of the aquatic ecosystem. In order to rapidly and sensitively detect the toxicity of heavy metals to microalgal photosynthesis, in this study, the effects of four typical toxic heavy metals, chromium (Cr(VI)), cadmium (Cd), mercury (Hg), and copper (Cu), on nine photosynthetic fluorescence parameters (φ_Po_, Ψ_Eo_, φ_Eo_, δ_Ro_, Ψ_Ro_, φ_Ro_, F_V_/F_O_, PI_ABS_, and S_m_) derived from the chlorophyll fluorescence rise kinetics (OJIP) curve of microalga *Chlorella pyrenoidosa*, were investigated based on the chlorophyll fluorescence induction kinetics technique. By analyzing the change trends of each parameter with the concentrations of the four heavy metals, we found that compared with other parameters, φ_Po_ (maximum photochemical quantum yield of photosystem II), F_V_/F_O_ (photochemical parameter of photosystem II), PI_ABS_ (photosynthetic performance index), and S_m_ (normalized area of the OJIP curve) demonstrated the same monotonic change characteristics with an increase in concentration of each heavy metal, indicating that these four parameters could be used as response indexes to quantitatively detect the toxicity of heavy metals. By further comparing the response performances of φ_Po_, F_V_/F_O_, PI_ABS_, and S_m_ to Cr(VI), Cd, Hg, and Cu, the results indicated that whether it was analyzed from the lowest observed effect concentration (LOEC), the influence degree by equal concentration of heavy metal, the 10% effective concentration (EC_10_), or the median effective concentration (EC_50_), the response sensitivities of PI_ABS_ to each heavy metal were all significantly superior to those of φ_Ro_, F_V_/F_O_, and S_m_. Thus, PI_ABS_ was the most suitable response index for sensitive detection of heavy metals toxicity. Using PI_ABS_ as a response index to compare the toxicity of Cr(VI), Cd, Hg, and Cu to *C. pyrenoidosa* photosynthesis within 4 h by EC_50_ values, the results indicated that Hg was the most toxic, while Cr(VI) toxicity was the lowest. This study provides a sensitive response index for rapidly detecting the toxicity of heavy metals to microalgae based on the chlorophyll fluorescence induction kinetics technique.

## 1. Introduction

As the source of life, water is an indispensable part of nature and human life. However, with the rapid development of socioeconomic events, industry, and agriculture, large quantities of heavy metals from anthropogenic activities, such as industrial emissions involving smelting and mining, extensive application of chemical fertilizers in agricultural production, automobile exhaust emissions, and garbage dumps in daily life, are discharged into the environment, resulting in serious heavy metal pollution problems in the aquatic environment [1,2,3]. Because heavy metals have the characteristics of non-degradability, bioaccumulation, and toxicity [4,5], the toxic effects of heavy metals on aquatic organisms in the aquatic environment have always been concerning.

In aquatic ecosystems, compared with other aquatic organisms, microalgae as kinds of planktonic photosynthetic organisms, are at the bottom of the aquatic food chain and are the main primary producers and energy converters [5,6]. As an important link in the material cycle and energy flow of aquatic ecosystems, microalgae play an important role in maintaining the normal structure and function of aquatic ecosystems [7]. Moreover, as a vital physiological process of microalgae, photosynthesis has important functions for aquatic ecosystems, which can provide material and energy sources for other organisms. As a result, microalgal photosynthesis is crucial for the normal primary production of the aquatic ecosystem [8]. However, many studies have reported that heavy metals have toxic effects on the photosynthesis of microalgae [1,8,9,10,11,12,13] by inhibiting the absorption of light energy, the transmission of photosynthetic electrons, and the conversion of photosynthetic energy [14,15,16], which will seriously affect the primary productivity of aquatic ecosystems and pose potential risks to the aquatic environment in severe cases. Therefore, rapid and sensitive detection of the toxicity of the heavy metals in water to the photosynthesis of microalgae is of great significance for evaluating the impacts of heavy metals on aquatic ecosystems and predicting their potential environmental risks.

The photosynthesis of plants is accompanied by the emission of chlorophyll fluorescence, and chlorophyll fluorescence is closely related to the photosynthesis state of plants [17,18]. Thus, the change in the photosynthesis state of plants can be determined by non-destructive measurement of chlorophyll fluorescence signals [19]. Based on this, the non-invasive and in vivo chlorophyll fluorescence induction kinetics technique has become a simple, rapid, reliable, and effective tool for analyzing the photosynthetic state [11,20,21,22] and photosystem II (PSII) behavior [1,16,20] of plants by conveniently and rapidly obtaining fluorescence information, including the chlorophyll fluorescence rise kinetics (OJIP) curve and diverse photosynthetic fluorescence parameters. In this way, the chlorophyll fluorescence induction kinetics technique has also become a favorable tool for rapid and on-site determination of pollutant toxicity to microalgal photosynthesis [23,24].

At present, although there are many photosynthetic fluorescence parameters derived from the OJIP curve, and some parameters have been widely used to evaluate the toxicity of heavy metals to microalgal photosynthesis based on the chlorophyll fluorescence induction kinetics technique, such as the maximum photochemical quantum yield of PSII (F_V_/F_M_) [12,20,21,25]. However, the response characteristics of different photosynthetic fluorescence parameters to heavy metals toxicity are not clear, and which parameter has the optimal response performance to the toxicity of heavy metals is still unknown. In this way, the use of an inappropriate response index will reduce the accuracy and sensitivity of fluorescence kinetics methods in detecting heavy metal toxicity. Therefore, it is extremely important to find an optimal response index to improve the accuracy and sensitivity of heavy metals toxicity detection based on the chlorophyll fluorescence induction kinetics technique.

In freshwater environments, the green alga *Chlorella pyrenoidosa* is a common unicellular freshwater microalga. Because of its wide distribution and vulnerability to toxic substances, *C. pyrenoidosa* has become an important indicative organism for detecting the toxicity of pollutants and evaluating the quality of the aquatic environment [15,21,26]. In this study, in order to rapidly and sensitively detect the toxicity of heavy metals to the photosynthesis of *C. pyrenoidosa*, the chlorophyll fluorescence induction kinetics technique was adopted to investigate the effects of four typical toxic heavy metals including chromium (Cr(VI)), cadmium (Cd), mercury (Hg), and copper (Cu) on the nine photosynthetic fluorescence parameters φ_Po_, Ψ_Eo_, φ_Eo_, δ_Ro_, Ψ_Ro_, φ_Ro_, F_V_/F_O_, PI_ABS_, and S_m_ derived from the OJIP curve. According to the change trends of each parameter with the concentrations of the four heavy metals, the parameters that could be used to quantitatively detect heavy metals toxicity were selected. Then by comparing their response performances to the toxicity of the four heavy metals, the most sensitive response index for detecting heavy metals toxicity was confirmed. On this basis, the optimal response index was used to compare the toxicity of Cr(VI), Cd, Hg, and Cu to the photosynthesis of *C. pyrenoidosa* at different exposure times during short-term stress within 4 h. This study will be helpful for the development of rapid and accurate detection methods for the toxicity of pollutants based on the chlorophyll fluorescence induction kinetics technique.

## 2. Materials and Methods

### 2.1. Algal Culture

The freshwater microalga *C. pyrenoidosa* used in this study was obtained from the Freshwater Algae Species Bank of the Institute of Hydrobiology, Chinese Academy of Sciences (Wuhan, China). After being autoclaved at 121 °C for 30 min, BG11 medium and 500 mL Erlenmeyer flasks were used to inoculate and culture *C. pyrenoidosa* [15,27]. *C. pyrenoidosa* was aseptically inoculated in 500 mL Erlenmeyer flasks containing sterile BG11 medium in a SW-CJ-1D ultra-clean workbench (Shangyu Aike Instrument Equipment Co., Ltd., Shaoxing, China). Then the inoculated algae samples were cultured in a MQD-B3G constant temperature incubator (Shanghai Minquan Instrument Co., Ltd., Shanghai, China) with white cold fluorescent tubes as the light source. The culture conditions were as follows: light intensity was 120 μmol m^−2^ s^−1^; light and dark cycle was 12 h:12 h; and culture temperature was (25 ± 1) °C [27]. The cell density of the algal culture was counted daily by an ECLIPSE Ni-U biological fluorescence microscope (Nikon Corporation, Tokyo, Japan). After being cultured for 3–4 days to enter the exponential growth phase, *C. pyrenoidosa* was used to carry out the exposure experiments of heavy metals.

### 2.2. Heavy Metals Exposure Experiments

Potassium dichromate (K_2_Cr_2_O_7_, CAS: 7778-50-9, purity ≥ 99.8%), cadmium chloride hemi (pentahydrate) (CdCl_2_·2.5H_2_O, CAS: 7790-78-5, purity ≥ 99.0%), mercury chloride (HgCl_2_, CAS: 7487-94-7, purity ≥ 99.5%), and copper sulfate pentahydrate (CuSO_4_·5H_2_O, CAS: 7758-99-8, purity ≥ 99.0%) were used as the sources of Cr(VI), Cd, Hg, and Cu for the heavy metals exposure experiments with *C. pyrenoidosa*, respectively. K_2_Cr_2_O_7_, CdCl_2_·2.5H_2_O, HgCl_2_, and CuSO_4_·5H_2_O were all of analytical grade and were all purchased from Sinopharm Chemical Reagent Co., Ltd. (Shanghai, China).

First, according to the preliminary experimental results, 0.2 M of Cr(VI) stock solution and 0.05 M of Cd, Hg, and Cu stock solutions were prepared by dissolving K_2_Cr_2_O_7_, CdCl_2_·2.5H_2_O, HgCl_2_, and CuSO_4_·5H_2_O in sterile BG11 medium, respectively. Then the stock solution of each heavy metal was further diluted with sterile BG11 medium to obtain a series of working solutions of each heavy metal with different concentrations. An iCAP PQ inductively coupled plasma mass spectrometer (ICP-MS, Thermo Fisher Scientific, Germany) was employed to further measure the accurate concentration of Cr(VI), Cd, Hg or Cu in each working solution, and the concentrations of heavy metals measured by ICP-MS were as follows: Cr(VI) concentrations in a series of Cr(VI) working solutions were 0.510, 0.969, 1.938, 3.927, 7.854, 15.708, 31.365, 62.781 and 125.562 mM; Cd concentrations in a series of Cd working solutions were 0.102, 0.204, 0.459, 0.918, 1.836, 3.621, 7.242, 14.535 and 29.070 mM; Hg concentrations in a series of Hg working solutions were 0.102, 0.255, 0.510, 0.765, 1.020, 1.275, 1.530, 1.785 and 2.040 mM; and Cu concentrations in a series of Cu working solutions were 0.204, 0.612, 0.816, 1.020, 1.224, 1.428, 1.632, 2.397, 3.213 and 6.426 mM.

Exposure experiments were performed according to standard OECD Guideline 201 [28] with minor modification. Prior to exposure to heavy metals, the *C. pyrenoidosa* culture was diluted with sterile BG11 medium to obtain an algal suspension with the expected cellular concentration (1 × 10^5^ cells mL^−1^). Then 1 mL of each heavy metal working solutions was added into aliquots of 50 mL of algal suspension; after that, each mixture was thoroughly shaken by hand to obtain a series of treatments of each heavy metal. The initial heavy metal concentrations of the treatments of each heavy metal were as follows: initial Cr(VI) concentrations of the Cr(VI) treatments were in the range of 0.010 to 2.462 mM; initial Cd concentrations of the Cd treatments ranged from 0.002 to 0.570 mM; initial Hg concentrations of the Hg treatments were in the range of 0.002 to 0.040 mM; and initial Cu concentrations of the Cu treatments ranged from 0.004 to 0.063 mM. Moreover, controls were prepared by adding 1 mL of sterile BG11 medium to aliquots of 50 mL of algal suspensions. Three replicates were performed for each treatment and the control. Then all the controls and treatments were placed in the incubator and cultured under the same culture conditions as described above. When the exposure times reached 1, 2, 3 and 4 h, the OJIP curve of each test alga sample was measured.

### 2.3. OJIP Curve Measurement

The chlorophyll fluorescence rise kinetics OJIP curve of each test alga sample was measured at room temperature according to Chen et al. (2016) [29] by an AquaPen AP110/C Handheld Algae Fluorescence Meter (Photon Systems Instruments, Czech Republic) with a blue light source of 455 nm. The saturation light pulse intensity was set as 1800 μmol (photons) m^−2^ s^−1^, and the measuring light pulse intensity was set as 0.027 μmol (photons) m^−2^ s^−1^. Prior to measurement, all the test algae samples were dark-adapted for 15 min to allow the PSII reaction centers (RCs) to open (re-oxidize) and the electron transport chain to be fully oxidized [14]. Fluorescence intensity data in a time span from 20 μs to 2 s were recorded with a varying sampling rate: from 20 to 610 μs, data were recorded per 10 μs; from 1 to 13.9 ms, data were recorded per 100 μs; from 15 to 89 ms, data were recorded per 1 ms; and from 90 ms to 2 s, data were recorded per 10 ms. The recorded 360 fluorescence intensity data before 1000 ms were used to draw the OJIP curve of each test alga sample.

### 2.4. Photosynthetic Fluorescence Parameters Acquisition

The following fluorescence data obtained from the original measured OJIP curves were used to calculate different photosynthetic fluorescence parameters: F_O_ is the initial fluorescence level of the OJIP curve, corresponding to the fluorescence intensity of O-step at 20 μs and representing the minimal fluorescence yield of the system; F_J_ is the fluorescence level of J-step, corresponding to the fluorescence intensity at about 2 ms; F_I_ is the fluorescence level of I-step, corresponding to the fluorescence intensity at about 20 ms; and F_M_ is defined as the maximal fluorescence intensity of the OJIP curve, which is equal to the fluorescence level of P-step [20]. Then the variable fluorescence F_V_ was calculated based on the above basic fluorescence intensity data according to Equation (1), which refers to the variation in fluorescence intensity between P-step and O-step of the OJIP curve.

F_V_ = F_M_ − F_O_ (1)

In addition, photosynthetic fluorescence parameters, including V_J_, V_I_, Mo, Area, φ_Po_, Ψ_Eo_, φ_Eo_, δ_Ro_, Ψ_Ro_, φ_Ro_, F_V_/F_O_, PI_ABS_, and S_m_, were calculated and obtained by the JIP-test based on F_O_, F_J_, F_I_, F_M_, and F_V_ values of the OJIP curve. The calculation equations and definitions of different parameters are shown in Table 1. Among them, φ_Po_ (also known as F_V_/F_M_) was the most frequently used parameter in detecting the toxicity of pollutants and diagnosing the photosynthetic state of plants under stress [25]. The K-S normality test was used to check the normality of the above parameters, and all data passed the normality test. Then φ_Po_, Ψ_Eo_, φ_Eo_, δ_Ro_, Ψ_Ro_, φ_Ro_, F_V_/F_O_, PI_ABS_, and S_m_ were used to analyze the impacts of heavy metals Cr(VI), Cd, Hg, and Cu on them, and φ_Po_, F_V_/F_O_, PI_ABS_, and S_m_ were further used as response indexes to detect the toxicity of Cr(VI), Cd, Hg, and Cu to the photosynthesis of *C. pyrenoidosa*, respectively.

### 2.5. Data Processing and Statistical Analysis

Statistical analyses of data were carried out using SPSS 19.0 software. The statistically significant differences between the control group and each treatment group were determined by using one-way analysis of variance (ANOVA) with the Tukey post-hoc multiple test. For the results, 0.01 ≤ *p* < 0.05 was marked with an asterisk (*) to indicate that the treatment group was significantly different from the control group, and *p* < 0.01 was marked with two asterisks (**) to indicate that there was a highly significant difference between the control group and the treatment group. In order to detect the toxicity of Cr(VI), Cd, Hg, and Cu to *C. pyrenoidosa* photosynthesis, when the response index of exposed *C. pyrenoidosa* was inhibited, the percentage inhibitions of the response index at different exposure times were calculated according to Equation (2):

I_t_ (%) = [(P_c−t_ − P_t−t_)/P_c−t_] × 100% (2)

where P_c−t_ is the response index of control at the exposure time of t; P_t−t_ is the response index of treatment at the exposure time of t; and I_t_ (%) is the percentage inhibition of response index at the exposure time of t. When the response index of exposed *C. pyrenoidosa* was promoted, the percentage promotions of the response index at different exposure times were calculated according to Equation (3):

F_t_ (%) = [(P_t−t_ − P_c−t_)/P_c−t_] × 100% (3)

where F_t_ (%) is the percentage promotion of response index at the exposure time of t.

For different response indexes of *C. pyrenoidosa*, the lowest observed effect concentration (LOEC) values of each heavy metal at different exposure times were determined by the lowest concentration of the heavy metal at which there was a significant difference between the treatment and the control [29]. All the concentration–response relationships between the response indexes of *C. pyrenoidosa* and each heavy metal were fitted using the logistic curve model [31], and the 10% effective concentration (EC_10_) values and median effective concentration (EC_50_) values of each heavy metal at different exposure times were calculated according to the fitted concentration–response curves [32].

## 3. Results

### 3.1. Influences of Four Heavy Metals on the OJIP Curve

**Figure 1:**
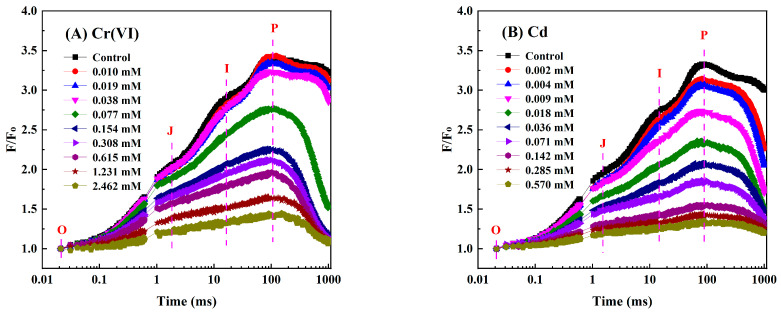

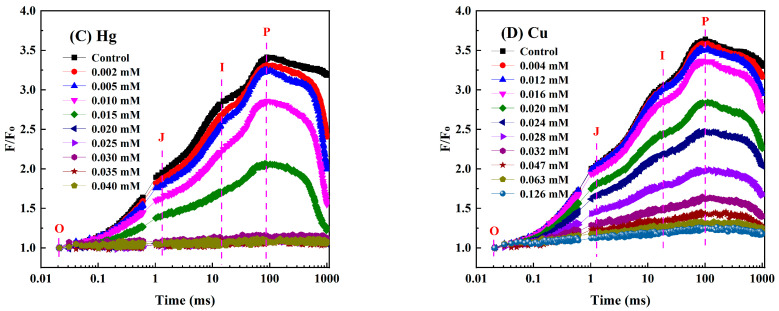
Normalized OJIP curves of *C. pyrenoidosa* exposed to different concentrations of heavy metals for 3 h: (**A**) Cr(VI), (**B**) Cd, (**C**) Hg, (**D**) Cu.

For the *C. pyrenoidosa* treated with Cr(VI), Cd, Hg, and Cu during a short-term exposure of 1–4 h, there was no further considerable change in the calculated inhibitions of photosynthetic activity comparing the 3 h long exposures to the 4 h long ones, so the 3 h long exposures are presented in this paper for detailed analyses. In order to accurately analyze the influences of Cr(VI), Cd, Hg, and Cu on the OJIP curve of *C. pyrenoidosa* and avoid the interferences of other factors, the directly measured OJIP curves of each treatment and control were first normalized with F_O_. When the exposure time was 3 h, the normalized OJIP curves (0.02–100 ms) of *C. pyrenoidosa* exposed to different concentrations of Cr(VI), Cd, Hg, and Cu are shown in Figure 1. As seen from Figure 1, for the normalized OJIP curve of unexposed *C. pyrenoidosa*, the fluorescence yield increased to a great extent from O-step (about 0.02 ms) to P-step (about 100 ms). In addition, there were also two obvious inflections between O-step and P-step, corresponding to J-step (about 2 ms) and I-step (about 20 ms), respectively. Because of the toxic effects of Cr(VI), Cd, Hg, and Cu on the photosynthesis of *C. pyrenoidosa*, the exposures to those four heavy metals induced significant changes in the normalized OJIP curve of *C. pyrenoidosa* compared with the control. That is, with an increase in heavy metal concentration, the fluorescence yield of the normalized OJIP curve gradually decreased, and the J-step and I-step in the normalized OJIP curve became less pronounced. Moreover, as the concentrations of heavy metals gradually increased, the fluorescence yield of P-step gradually approximated that of O-step, so that the photosynthetic fluorescence parameter F_V_/F_O_ gradually decreased. When *C. pyrenoidosa* was exposed to Cr(VI), Cd, Hg, and Cu for 1, 2, and 4 h, the normalized OJIP curve had the same change characteristics as those of the 3 h exposures.

### 3.2. Changes of Different Parameters with Heavy Metals Concentrations

**Figure 2:**
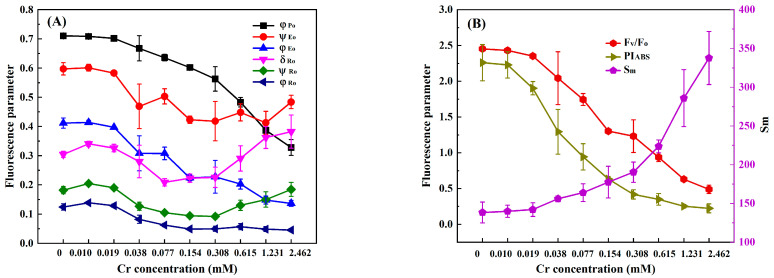
Parameter values of *C. pyrenoidosa* exposed to different concentrations of Cr(VI) for 3 h: (**A**) φ_Po_, Ψ_Eo_, φ_Eo_, δ_Ro_, Ψ_Ro_ and φ_Ro_; (**B**) F_V_/F_O_, PI_ABS_, and S_m_. Symbols and error bars represent the average values and standard deviations of triplicates, respectively.

**Figure 3:**
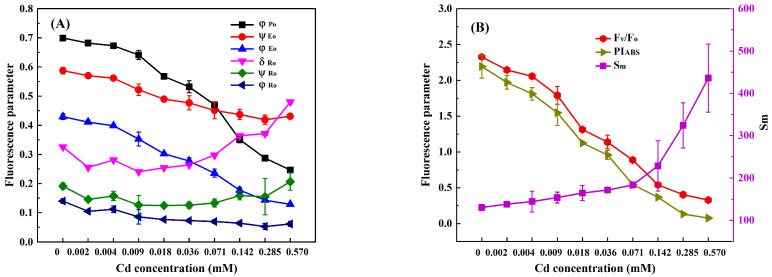
Parameter values of *C. pyrenoidosa* exposed to different concentrations of Cd for 3 h: (**A**) φ_Po_, Ψ_Eo_, φ_Eo_, δ_Ro_, Ψ_Ro_ and φ_Ro_; (**B**) F_V_/F_O_, PI_ABS_, and S_m_. Symbols and error bars represent the average values and standard deviations of triplicates, respectively.

**Figure 4:**
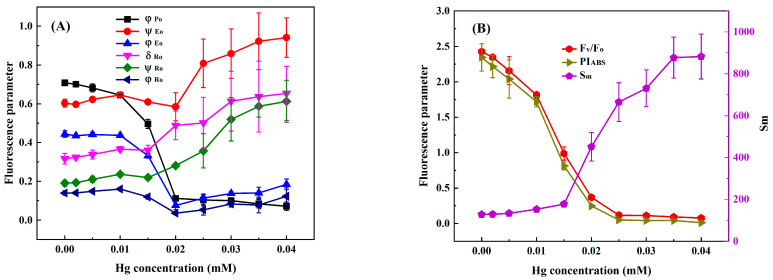
Parameter values of *C. pyrenoidosa* exposed to different concentrations of Hg for 3 h: (**A**) φ_Po_, Ψ_Eo_, φ_Eo_, δ_Ro_, Ψ_Ro_ and φ_Ro_; (**B**) F_V_/F_O_, PI_ABS_, and S_m_. Symbols and error bars represent the average values and standard deviations of triplicates, respectively.

**Figure 5:**
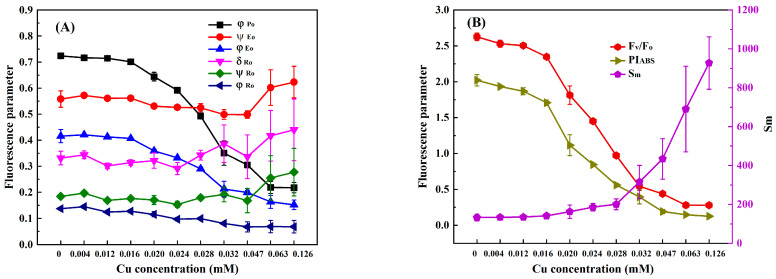
Parameter values of *C. pyrenoidosa* exposed to different concentrations of Cu for 3 h: (**A**) φ_Po_, Ψ_Eo_, φ_Eo_, δ_Ro_, Ψ_Ro_ and φ_Ro_; (**B**) F_V_/F_O_, PI_ABS_, and S_m_. Symbols and error bars represent the average values and standard deviations of triplicates, respectively.

In order to select suitable response indexes for detecting heavy metals toxicity based on the chlorophyll fluorescence induction kinetics technique, we analyzed the changes in nine photosynthetic fluorescence parameters, φ_Po_, Ψ_Eo_, φ_Eo_, δ_Ro_, Ψ_Ro_, φ_Ro_, F_V_/F_O_, PI_ABS_, and S_m_, derived from the OJIP curve of *C. pyrenoidosa* with the concentrations of Cr(VI), Cd, Hg, and Cu. At the exposure time of 3 h, the values of φ_Po_, Ψ_Eo_, φ_Eo_, δ_Ro_, Ψ_Ro_, φ_Ro_, F_V_/F_O_, PI_ABS_, and S_m_ of *C. pyrenoidosa* exposed to different concentrations of Cr(VI), Cd, Hg, and Cu are shown in Figure 2, Figure 3, Figure 4 and Figure 5. It can be seen that as the concentration of each heavy metal gradually increased, the values of φ_Po_, F_V_/F_O_, and PI_ABS_ monotonically decreased, and the value of S_m_ monotonically increased. However, the changes in the other five parameters with the concentrations of the four heavy metals were different from those of φ_Po_, F_V_/F_O_, PI_ABS_, and S_m_. For example, although the parameters φ_Eo_ and φ_Ro_ gradually decreased with an increase in Cr(VI), Cd, Hg, and Cu concentrations, when the Hg concentration was greater than 0.020 mM, the values of φ_Eo_ and φ_Ro_ slightly increased instead. Moreover, as the concentrations of Cr(VI), Cd, Hg, and Cu increased, both δ_Ro_ and Ψ_Ro_ showed a trend of gradually decreasing first and then slowly increasing. In addition, although the parameter Ψ_Eo_ gradually decreased with the increase in Cr(VI) and Cd concentrations, there was little change in Ψ_Eo_ within the lower concentration ranges of Hg and Cu (Hg ≤ 0.020 mM and Cu ≤ 0.047 mM); then, when the concentration of Hg was greater than 0.02 mM and the concentration of Cu was greater than 0.047 mM, Ψ_Eo_ demonstrated a gradually increasing trend. When *C. pyrenoidosa* was exposed to Cr(VI), Cd, Hg, and Cu for 1, 2, and 4 h, the change characteristics of each parameter with four heavy metal concentrations were the same as those of the 3 h exposures. These results indicated that when the heavy metals were different, the change trends of Ψ_Eo_, φ_Eo_, δ_Ro_, Ψ_Ro_, and φ_Ro_ with heavy metal concentration may be inconsistent. In contrast, all the parameters φ_Po_, F_V_/F_O_, PI_ABS_, and S_m_ showed monotonic changes with heavy metal concentration, indicating that these four parameters were suitable for quantitative detection of the toxicity of heavy metals.

### 3.3. Comparison of Response Performances of φ_Po_, F_V_/F_O_, PI_ABS_ and S_m_ to Four Heavy Metals Toxicity

In view of the fact that all four heavy metals Cr(VI), Cd, Hg, and Cu had important impacts on the φ_Po_, F_V_/F_O_, PI_ABS_, and S_m_ values of *C. pyrenoidosa*, and all four parameters had monotonic concentration-dependences with each heavy metal, in order to select a suitable response index for rapid and sensitive detection of the toxicity of heavy metals to the photosynthesis of *C. pyrenoidosa*, the response performances of φ_Po_, F_V_/F_O_, PI_ABS_, and S_m_ to the four heavy metals’ toxicity were compared.

**Figure 6:**
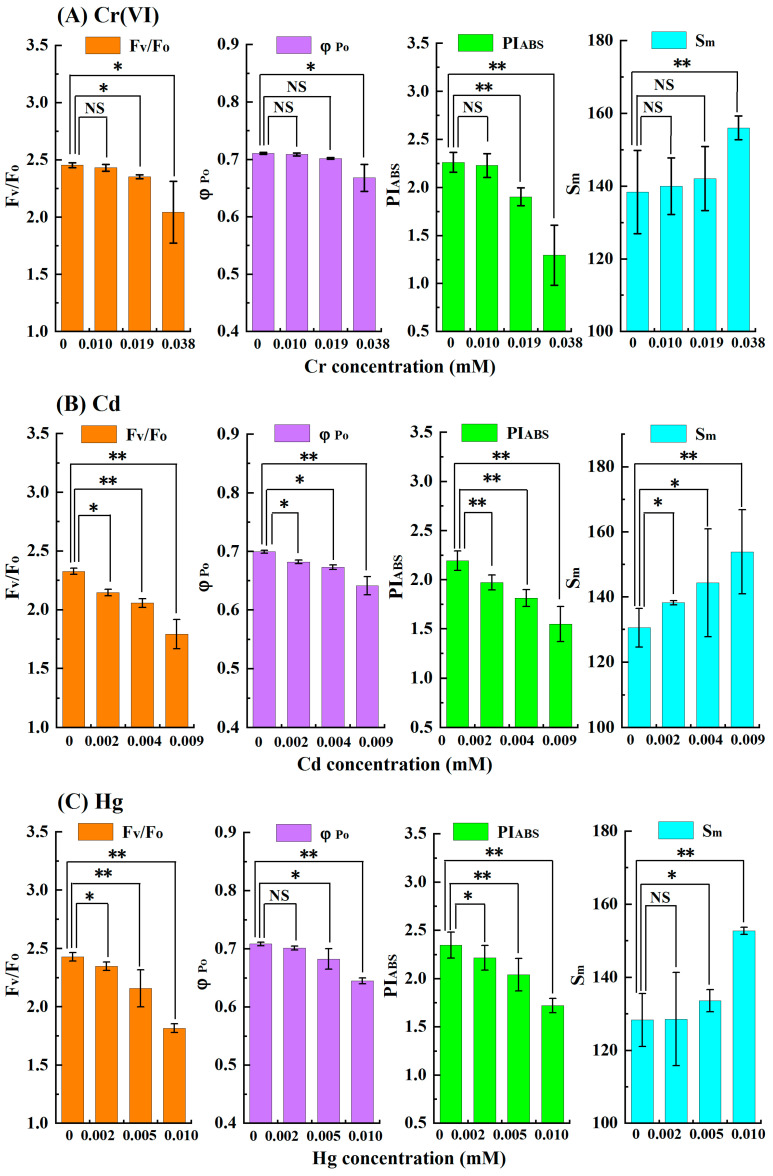

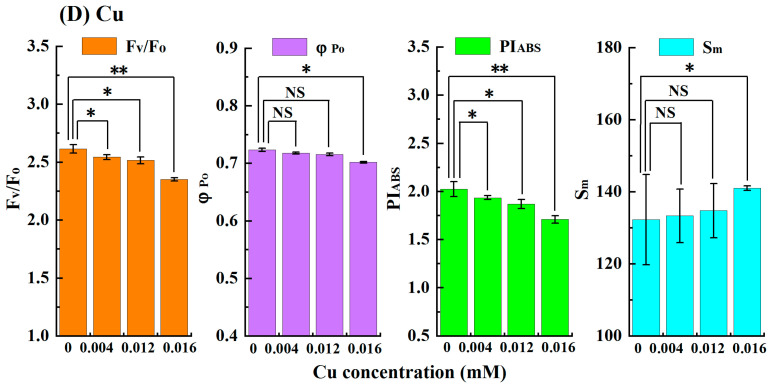
Significant differences in φ_Po_, F_V_/F_O_, PI_ABS_, and S_m_ for *C. pyrenoidosa* exposed to a low concentration of heavy metal for 3 h and the control: (**A**) Cr(VI), (**B**) Cd, (**C**) Hg, (**D**) Cu. An asterisk (*) indicates a significant difference between the treatment group and the control group at 0.01 ≤ *p* < 0.05; and two asterisks (**) indicate a highly significant difference between the treatment group and the control group at *p* < 0.01. “NS” indicates that there is no significant difference between the treatment group and the control group. Columns and error bars represent the average values and standard deviations of triplicates, respectively.

First, the response sensitivities of φ_Po_, F_V_/F_O_, PI_ABS_, and S_m_ to low concentrations of Cr(VI), Cd, Hg, and Cu were compared. At the exposure time of 3 h, the significant differences in φ_Po_, F_V_/F_O_, PI_ABS_, and S_m_ between the low concentration of heavy metal treatments and the control are shown in Figure 6. It can be seen that when the concentration of Cr(VI) reached 0.019 mM, there was a significant difference in F_V_/F_O_ (*p* = 0.012) and a highly significant difference in PI_ABS_ (*p* = 0.008) between the treatment and the control, while the φ_Po_ and S_m_ parameters of the treatment had no significant differences from those of the control. For 0.020 mM Cd treatment, compared with the control, PI_ABS_ showed a highly significant difference (*p* = 0.009), while F_V_/F_O_, φ_Po_ and S_m_ only showed significant differences (*p* = 0.012, 0.042, and 0.046, respectively). Similarly, when the concentrations of Hg and Cu were as low as 0.002 and 0.004 mM, respectively, the F_V_/F_O_ and PI_ABS_ of the treatments were significantly different from those of the control (for the Hg treatment, *p* = 0.032 (F_V_/F_O_) and 0.045 (PI_ABS_); for the Cu treatment, *p* = 0.036 (F_V_/F_O_) and 0.041 (PI_ABS_)), while there were no significant differences in φ_Po_ and S_m_ between the treatments and the control. These results indicated that when PI_ABS_ and F_V_/F_O_ were used as test endpoints, the LOEC values of the four heavy metals were significantly lower than the values determined using φ_Po_ and S_m_ as test endpoints. Moreover, compared with F_V_/F_O_, PI_ABS_ of the same low-concentration heavy metal treatment showed a more significant difference from that of the control. When the exposure times were 1, 2, and 4 h, the comparison results of the different parameters in response to low concentrations of the heavy metal were similar to those of the 3 h exposures. Thus, the response sensitivity of PI_ABS_ to low concentrations of heavy metals was significantly higher than that of F_V_/F_O_, φ_Po_, and S_m_.

**Figure 7:**
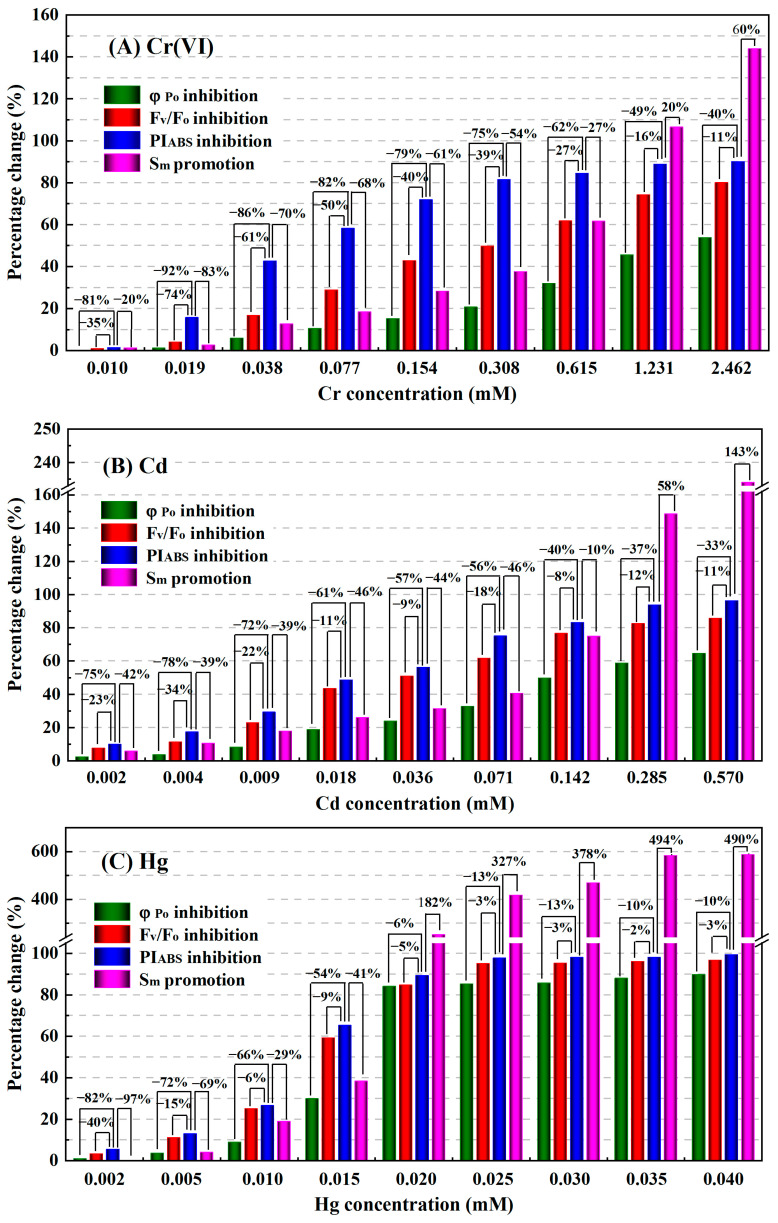

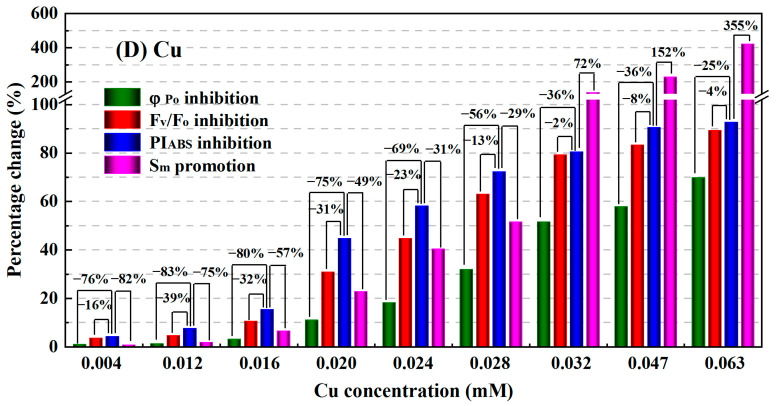
Percentage changes of φ_Po_, F_V_/F_O_, PI_ABS_, and S_m_ of *C. pyrenoidosa* exposed to different concentrations of heavy metal for 3 h: (**A**) Cr(VI), (**B**) Cd, (**C**) Hg, (**D**) Cu.

Second, for each treatment exposed for the same time, the influence degrees of φ_Po_, F_V_/F_O_, PI_ABS_, and S_m_ of *C. pyrenoidosa* by the same concentration of heavy metal were also compared, and the results at the exposure time of 3 h are shown in Figure 7. We can see that regardless of whether *C. pyrenoidosa* was exposed to Cr(VI), Cd, Hg, or Cu, when the heavy metal concentration was the same, all the percentage changes of PI_ABS_ were significantly greater than those of φ_Po_, F_V_/F_O_ and S_m_. For example, when the concentration of Cr(VI), Cd, Hg, and Cu increased from 0.010 to 2.462 mM, from 0.002 to 0.570 mM, from 0.002 to 0.040 mM, and from 0.004 to 0.063 mM, respectively, the percentage inhibition of PI_ABS_ increased correspondingly from 1.45% to 90.09%, from 10.05% to 96.44%, from 5.57% to 99.49%, and from 4.35% to 92.7%, respectively; and the corresponding percentage inhibition of F_V_/F_O_ increased from 0.92% to 80.06%, from 7.75% to 85.91%, from 3.33% to 96.80%, and from 3.65% to 89.30%, respectively; while the corresponding percentage inhibition of φ_Po_ only increased from 0.26% to 53.86%, from 2.46% to 64.72%, from 0.99% to 89.86%, and from 1.03% to 69.76%, respectively. In the concentration ranges of heavy metals used in this study, the percentage inhibitions of φ_Po_ induced by Cr(VI), Cd, Hg, and Cu were 40–92%, 33–78%, 6–82%, and 25–83% lower than those of PI_ABS_, with average reductions of 72%, 57%, 36% and 59%, respectively; and the percentage inhibitions of F_V_/F_O_ caused by Cr(VI), Cd, Hg, and Cu decreased by averages of 39%, 16%, 9% and 18% compared with those of PI_ABS_, respectively. Thus, the influence degrees of PI_ABS_ for equal concentrations of Cr(VI), Cd, Hg, and Cu were significantly higher than those of F_V_/F_O_ and φ_Po_. For the same heavy metal treatment, although the percentage changes of S_m_ were significantly higher than the percentage inhibitions of PI_ABS_ within the range of higher concentrations of heavy metals (such as Cr(VI) ≥ 1.231 mM, Cd ≥ 0.285 mM, Hg ≥ 0.020 mM, and Cu ≥ 0.031 mM), when the concentrations of heavy metals were lower, the percentage changes of S_m_ were also lower than the percentage inhibitions of PI_ABS_. When the exposure times were 1, 2, and 4 h, the comparison results of the influence degrees of φ_Po_, F_V_/F_O_, PI_ABS_, and S_m_ for the same concentrations of Cr(VI), Cd, Hg, and Cu were the same as those of the 3 h exposures. Therefore, the response sensitivity of PI_ABS_ to the same concentration of heavy metal was significantly better than those of φ_Po_, F_V_/F_O_, and S_m_.

In addition, the EC_10_ and EC_50_ values of Cr(VI), Cd, Hg, and Cu calculated according to φ_Po_, F_V_/F_O_, PI_ABS_, and S_m_ were further compared when *C. pyrenoidosa* was exposed for the same time, and when the exposure time was 3 h, the results are shown in Table 2. It can be seen that when PI_ABS_ was used as the test endpoint of *C. pyrenoidosa* to detect the toxicity of heavy metals, the EC_50_ values of Cr(VI), Cd, Hg, and Cu were 0.054, 0.023, 0.013 and 0.022 mM, respectively, which were significantly lower than the EC_50_ values of these four heavy metals calculated based on φ_Po_, F_V_/F_O_, and S_m_. For Cr(VI), Cd, Hg, and Cu, compared to the EC_50_ values calculated according to PI_ABS_, the EC_50_ values calculated according to φ_Po_ were 33.95, 6.98, 1.25, and 1.78 times those values; the EC_50_ values calculated according to F_V_/F_O_ were 5.04, 1.36, 1.04, and 1.14 times those values; and the EC_50_ values calculated according to S_m_ were 7.45, 3.84, 1.09,and 1.31 times those values, respectively. Similarly, the EC_10_ values of each heavy metal calculated according to φ_Po_, F_V_/F_O_, and S_m_ were also significantly higher than those values calculated according to PI_ABS_. When the exposure time was 1, 2, and 4 h, the comparison results of the EC_10_ and EC_50_ values of Cr(VI), Cd, Hg, and Cu calculated according to φ_Po_, F_V_/F_O_, PI_ABS_, and S_m_ were similar to those of the 3 h exposures. So compared with φ_Po_, F_V_/F_O_, and S_m_, PI_ABS_ could be used as a response index to more sensitively detect the toxicity of Cr(VI), Cd, Hg, and Cu to the photosynthesis of *C. pyrenoidosa*.

Consequently, for *C. pyrenoidosa* exposed to Cr(VI), Cd, Hg, and Cu, whether it was analyzed from the LOEC values, the influence degrees by equal concentrations of the heavy metal, the EC_10_ or EC_50_ values, and the response sensitivities of PI_ABS_ to the toxicity of each heavy metal were all significantly superior to those of φ_Po_, F_V_/F_O_, and S_m_. So PI_ABS_ was a more suitable response index for rapidly and sensitively detecting the toxicity of heavy metals to the photosynthesis of *C. pyrenoidosa*.

### 3.4. Comparison of the Toxicity of Four Heavy Metals to the Photosynthesis of C. pyrenoidosa during Short-Term Stress within 4 h by PI_ABS_

**Figure 8:**
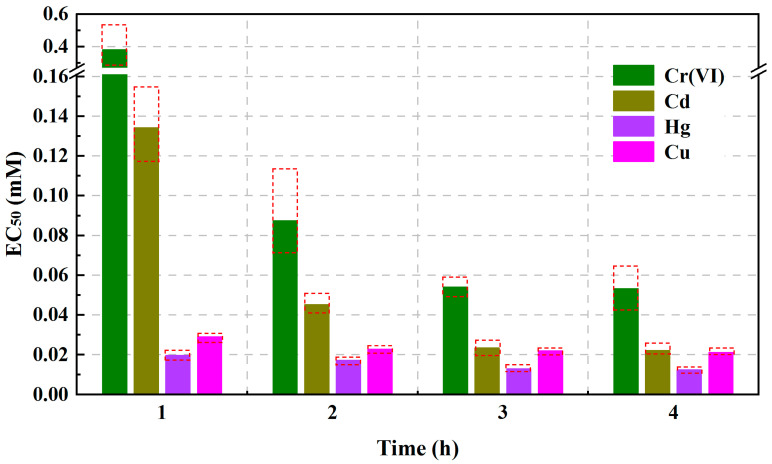
EC_50_ values of Cr(VI), Cd, Hg, and Cu at exposure times of 1, 2, 3 and 4 h using PI_ABS_ as the response index. The upper and lower borders of the red dashed boxes represent the 95% confidence interval of the EC_50_ value.

Among the four parameters φ_Po_, F_V_/F_O_, PI_ABS_, and S_m_, PI_ABS_, which had the most sensitive response performance, was used as a test endpoint to evaluate and compare the toxicity of Cr(VI), Cd, Hg, and Cu to the photosynthesis of *C. pyrenoidosa* during short-term stress within 4 h, and the EC_50_ values of the four heavy metals at the exposure times of 1, 2, 3, and 4 h are shown in Figure 8. As can be seen from Figure 8, within 4 h, the EC_50_ values of all four heavy metals decreased with an increase in exposure time. This result indicated that the toxic effects of the four heavy metals on the photosynthesis of *C. pyrenoidosa* were time-dependent during short-term stress. Although the EC_50_ values of the four heavy metals changed with the exposure time within 4 h, when the exposure time was the same, all the orders of EC_50_ values of the four heavy metals followed Hg < Cu < Cd < Cr(VI). Thus, during the short-term stress within 4 h, the toxicity order of the four heavy metals to *C. pyrenoidosa* photosynthesis was Hg > Cu > Cd > Cr(VI). Those results demonstrated that in a freshwater environment, among the four heavy metals, the photosynthesis of *C. pyrenoidosa* was most vulnerable to the toxicity of Hg, while the toxicity of Cr(VI) had a weaker impact on the photosynthesis of *C. pyrenoidosa*.

## 4. Discussion

In the present study, by analyzing the change characteristics of the normalized OJIP curve of *C. pyrenoidosa* exposed to heavy metals Cr(VI), Cd, Hg, and Cu compared with the control, we found that all four heavy metals had important effects on the normalized OJIP curve of *C. pyrenoidosa*, resulting in a reduction in fluorescence yield of the entire curve, which was consistent with the results of previous reports that heavy metals could decrease the fluorescence yields of the OJIP curves of *Chlorella valgaris* [16], *Scenedesmus incrassatululus* [18], *Scenedesmus obliquus* [33], and *Spirodela polyrhiza* [1].

Cr(VI), Cd, Hg, and Cu induced changes in the OJIP curve of *C. pyrenoidosa* because Cr(VI), Cd, Hg, and Cu, as toxic heavy metal pollutants, inhibited the photosynthetic activity of *C. pyrenoidosa*. The OJIP curve contained abundant information about the photochemical reaction of PSII, thus reflecting the changes in the photosynthetic state of *C. pyrenoidosa*. According to previous reports, PSII was the major target site of toxic heavy metals Cr(VI), Cd, Hg, and Cu [16,34,35]. When algal cells were exposed to Cr(VI), Cd, Hg, or Cu, these four heavy metals might reduce the light-capturing performance of light-harvesting antenna complexes (LHCs) and inhibit the electron transport of PSII via Q_A_, Q_B_, and the plastoquinone pool, thereby causing a reduction in the active PSII RCs and a decrease in the quantum yield of PSII [5,14,16,18,36]. Moreover, the nine photosynthetic fluorescence parameters φ_Po_, Ψ_Eo_, φ_Eo_, δ_Ro_, Ψ_Ro_, φ_Ro_, F_V_/F_O_, PI_ABS_, and S_m_ were all calculated based on the fluorescence information from the OJIP curve. Although these nine parameters were all affected by Cr(VI), Cd, Hg, and Cu, because they had different photosynthetic physiological meanings and represented the information of different photosynthetic stages, their response characteristics to heavy metals toxicity were inconsistent. If a parameter could be used as a response index for quantitative detection of heavy metals toxicity, for different heavy metals, it should have the same monotonic change characteristics with the change in heavy metal concentration. In this study, the parameters φ_Po_, Ψ_Eo_, φ_Eo_, δ_Ro_, Ψ_Ro_, φ_Ro_, F_V_/F_O_, PI_ABS_, and S_m_ were all affected by Cr(VI), Cd, Hg, and Cu. However, for different heavy metals, only φ_Po_, F_V_/F_O_, PI_ABS_, and S_m_ showed the same monotonic change trend with the increase in each heavy metal concentration, while the change trends of Ψ_Eo_, φ_Eo_, δ_Ro_, Ψ_Ro_, and φ_Ro_ with an increase in each heavy metal concentration were neither monotonic nor completely consistent. Therefore, parameters φ_Po_, F_V_/F_O_, PI_ABS_, and S_m_ could be used as test endpoints to quantitatively detect heavy metals toxicity, while parameters Ψ_Eo_, φ_Eo_, δ_Ro_, Ψ_Ro_ and φ_Ro_ were not applicable.

In order to select an optimal response index for rapidly and sensitively detecting the toxicity of heavy metals to the photosynthesis of *C. pyrenoidosa*, the response sensitivities of φ_Po_, F_V_/F_O_, PI_ABS_, and S_m_ to the toxicity of Cr(VI), Cd, Hg, and Cu were compared. We found that among the four photosynthetic activity parameters, no matter whether compared in terms of the LOEC values, the influence degrees for equal concentrations of heavy metal, the EC_10_ values, or the EC_50_ values, the response sensitivities of PI_ABS_ to the toxicity of Cr(VI), Cd, Hg, and Cu were all better than those of φ_Po_, F_V_/F_O_, and S_m_. Among them, parameter φ_Po_ (also known as F_V_/F_M_) represented the maximum photochemical quantum yield of PSII and is a frequently and widely used photosynthetic fluorescence parameter in the assessment of various pollutants’ toxicity. By comparison, we verified that φ_Po_ did not have very good sensitivity in response to the toxicity of heavy metal. If φ_Po_ is used to evaluate the toxic effects of heavy metals on the photosynthesis of microalgae, the degrees of toxic effect will be underestimated. The photosynthetic activity parameter PI_ABS_ had a more sensitive response performance to heavy metals, which was a more appropriate response index for quantitatively detecting the toxicity of heavy metals to *C. pyrenoidosa* using the chlorophyll fluorescence induction kinetics technique. The studies of Xin et al. (2020) [37] and Hu et al. (2018) [38] also indicated that PI_ABS_ (the performance index for energy conservation from photons absorbed by PSII to the reduction of intersystem electron acceptors) was more sensitive than φ_Po_ (also known as F_V_/F_M_) and F_V_/F_O_ in reflecting the impact of the heavy metal Cd and temperature on the photosynthesis of aquatic macrophyte *Pontederia cordata* and cotton seedlings. Therefore, the result of our study was in good agreement with the reports of Xin et al. (2020) and Hu et al. (2018). However, it is currently uncertain whether the response sensitivities of PI_ABS_ to other types of toxic pollutants are also superior to those of φ_Po_, F_V_/F_O_, and other photosynthetic fluorescence parameters. Therefore, we will conduct detailed research on the response characteristics of different photosynthetic fluorescence parameters to other toxic pollutants in the future.

In addition, we used PI_ABS_ as a test endpoint to compare the toxicity of Cr(VI), Cd, Hg, and Cu to the photosynthesis of *C. pyrenoidosa* during short-term stress within 4 h. We found that among the four heavy metals, Hg was the most toxic to *C. pyrenoidosa*, followed by Cu and Cd, while the toxicity of Cr(VI) was the lowest. Rocchetta et al. (2009) reported that treatment with Cu led to clearer and stronger effects on the photochemistry of *Euglena gracilis* than the treatment with Cr(VI) [39]. Eom et al. (2021) assessed and compared the toxicity of different heavy metals to *Chlorella vulgaris* by oxygen evolution, and the results indicated that the toxicity of Hg was higher than Cd according to 18 h EC_50_ values [40]. Liu et al. (2011) compared the toxicity of Cu and Cd on the motility of the two marine microalgae *Isochrysis galbana* and *Tetraselmis chui*, and their results demonstrated that the toxic effect of Cu on the motility of the two species was greater than that of Cd [41]. Tonon et al. (2018) analyzed the tolerance of *Gracilaria tenuistipitata* to Cd and Cu by observing photosynthesis, and they found that the toxicity of Cu was also higher than Cd toxicity to *Gracilaria tenuistipitata* according to 6 days EC_50_ [42]. Although the test organisms, toxicity test endpoints, and exposure times used in these previous studies were different from the present study, the toxicity ranking of the four heavy metals to the test organisms was in good agreement with our results. In the present study, Cr(VI) demonstrated the weakest toxicity compared with Cd, Hg, and Cu, which may be due to the fact that the PSII is not the main site where this metal exerts its action.

Moreover, we also found that although the response sensitivities of PI_ABS_ to Cr(VI), Cd, Hg, and Cu were better than those of φ_Po_, F_V_/F_O_, and S_m_, the EC_50_ values of Cr(VI), Cd, Hg, and Cu determined based on PI_ABS_ were still not very low during the short-term stress within 4 h. For example, Reis et al. (2021) [8] and Rocha et al. (2021) [9] reported that the 96 h EC_50_ value of Cd inhibiting the growth of *Raphidocelis subcapitata* was 0.67 μM, and the 72 h EC_50_ value of Cu inhibiting the growth of *Selenastrum gracile* was 0.06 μM. Because the toxicity of heavy metals is time-dependent, the short exposure time (within 4 h) in this study may be the reason why the EC_50_ values of Cr(VI), Cd, Hg, and Cu obtained based on PI_ABS_ were not very low. The low toxicity determined by PI_ABS_ in the hour time-scale may hide stronger effects (such as genotoxicity and growth toxicity) after longer exposures. Therefore, in practical application, during short-term exposure at an hour time scale, PI_ABS_ may be more suitable for the detection and evaluation of the toxicity of industrial wastewater and other polluted water containing higher concentrations of pollutants. Furthermore, algal population is a key indicator to evaluate the aquatic ecological environment. At present, although using the biomass or growth rate of algal population as a test endpoint to detect the toxicity of pollutants is a commonly used toxicity test method, these response indicators usually require longer exposure time. Because these test endpoints have difficulty in achieving rapid detection of the toxicity of pollutants or contaminated water, they have certain limitations in terms of the monitoring and management of the water environment and the emergency detection of sudden water pollution events. In contrast, the photosynthetic activity parameter PI_ABS_ selected in this study has a rapid response characteristic to heavy metals. Thus, establishing the relationship between PI_ABS_ and algal biomass or the growth rate of the algal population is of great significance for improving the practical application performance of PI_ABS_. Therefore, in our follow-up work, we plan to carry out detailed and systemic research on the relationship between the inhibition degree of PI_ABS_ by heavy metals after short-term exposure and the inhibition degree of algal biomass or growth rate by heavy metals after long-term exposure and try to construct their relationship models in order to infer the long-term impacts of heavy metals on the algae population based on the monitoring of PI_ABS_ over a short time. We hope that our subsequent works will provide methods with more practical application value for the evaluation of aquatic environment quality and the prediction of aquatic environmental risk.

## 5. Conclusions

In summary, the toxic effects of heavy metals Cr(VI), Cd, Hg, and Cu on the photosynthesis of *C. pyrenoidosa* induced significant changes in the OJIP curves of *C. pyrenoidosa*. As a result, Cr(VI), Cd, Hg, and Cu had significant impacts on photosynthetic fluorescence parameters φ_Po_, Ψ_Eo_, φ_Eo_, δ_Ro_, Ψ_Ro_, φ_Ro_, F_V_/F_O_, PI_ABS_, and S_m_ derived from the OJIP curve. Among the nine parameters, φ_Po_, F_V_/F_O_, PI_ABS_, and S_m_ showed the same monotonic change trends with the increase in each heavy metal concentration and were suitable for quantitatively detecting the toxicity of heavy metals. On the contrary, Ψ_Eo_, φ_Eo_, δ_Ro_, Ψ_Ro_, and φ_Ro_ were not suitable for assessment of heavy metals toxicity because their change trends with the concentration of each heavy metal were not completely consistent or not monotonic. Among the four parameters φ_Po_, F_V_/F_O_, PI_ABS_, and S_m_, the response sensitivities of PI_ABS_ to the four heavy metals were all better than those of φ_Po_, F_V_/F_O_, and S_m_, verifying that PI_ABS_ was a more sensitive response index than φ_Po_, F_V_/F_O_, and S_m_ in quantitatively detecting the toxicity of heavy metals. During short-term stress within 4 h, using PI_ABS_ as a response index to compare the toxicity of Cr(VI), Cd, Hg, and Cu to the photosynthesis of *C. pyrenoidosa*, the toxicity order of the four heavy metals was Hg > Cu > Cd > Cr(VI). This study provides an important basis and a sensitive response index for rapidly detecting heavy metals toxicity in water based on the chlorophyll fluorescence induction kinetics technique.

## Figures and Tables

**Table 1:** Calculations and definitions of different photosynthetic fluorescence parameters [30].

Parameter and Equation	Definition
V_J_ = (F_J_ − F_O_)/(F_M_ − F_O_)	Relative variable fluorescence at J-step
V_I_ = (F_I_ − F_O_)/(F_M_ − F_O_)	Relative variable fluorescence at I-step
M_O_ = 4·(F_300μs_ − F_O_)/(F_M_ − F_O_)	Approximate value of the initial slope of the relative variable fluorescence curve
Area	Area between the OJIP curve and the line F = F_M_
F_V_/F_O_	PSII photochemical parameter
S_m_ = Area/F_V_	Normalized area of the OJIP curve
φ_Po_ = 1 − F_O_/F_M_ = F_V_/F_M_	Maximum quantum yield of primary PSII photochemistry
φ_Eo_ = φ_Po_·(1-V_J_) = 1 − F_J_/F_M_	Quantum yield of the electron transport flux from Q_A_ to Q_B_
φ_Ro_ = φ_Po_·(1-V_I_) = 1 − F_I_/F_M_	Quantum yield of the electron transport flux until the PSI electron acceptors
Ψ_Eo_ = 1 − V_J_	Efficiency with which a PSII trapped electron is transferred from Q_A_ to Q_B_
Ψ_Ro_ = 1 − V_I_	Efficiency with which a PSII trapped electron is transferred until PSI acceptors
δ_Ro_ = (1 − V_I_)/(1-V_J_)	Efficiency with which an electron from Q_B_ is transferred until PSI acceptors
PI_ABS_ = [γ_RC_/(1 − γ_RC_)]·[φ_Po_/(1 − φ_Po_)]·[Ψ_Eo_/(1 − Ψ_Eo_)]	Performance index for energy conservation from photons absorbed by PSII antenna to the reduction of Q_B_

**Table 2:** Three-hour EC_10_ and EC_50_ values and their confidence intervals (95%) of four heavy metals with φ_Po_, F_V_/F_O_, PI_ABS_, and S_m_ as response indexes.

Heavy Metal	3 h EC_10_ (mM)				3 h EC_50_ (mM)			
	φ_Po_	F_V_/F_O_	PI_ABS_	S_m_	φ_Po_	F_V_/F_O_	PI_ABS_	S_m_
Cr(VI)	0.086 (0.064–0.116)	0.024 (0.019–0.030)	0.014 (0.013–0.015)	0.043 (0.042–0.044)	1.835 (1.548–2.309)	0.272 (0.223–0.336)	0.054 (0.049–0.059)	0.403 (0.322–0.491)
Cd	0.010 (0.008–0.015)	0.003 (0.002–0.004)	0.002 (0.002–0.003)	0.004 (0.003–0.005)	0.164 (0.127–0.212)	0.032 (0.025–0.041)	0.023 (0.020–0.027)	0.090 (0.089–0.091)
Hg	0.013 (0.011–0.014)	0.007 (0.006–0.008)	0.005 (0.004–0.006)	0.010 (0.009–0.011)	0.016 (0.016–0.017)	0.013 (0.013–0.014)	0.013 (0.012–0.014)	0.014 (0.011–0.017)
Cu	0.019 (0.015–0.023)	0.015 (0.011–0.017)	0.013 (0.010–0.014)	0.019 (0.017–0.022)	0.039 (0.034–0.046)	0.025 (0.024–0.027)	0.022 (0.021–0.023)	0.029 (0.027–0.031)

## Data Availability

The data presented in this article and data for 1, 2 and 4 h exposures are available on request from the corresponding authors.
